# Bilateral facial palsy after COVID-19 vaccination

**DOI:** 10.1007/s10072-022-05982-4

**Published:** 2022-04-01

**Authors:** Valentina Andreozzi, Beatrice D’arco, Pasquale Pagliano, Antonella Toriello, Paolo Barone

**Affiliations:** 1 Neurology Unit, Department of Medicine and Surgery, “Scuola Medica Salernitana”, University of Salerno, Fisciano, Italy; 2 Infective Disease Unit, Department of Medicine and Surgery, Scuola Medica Salernitana”, University of Salerno, Fisciano, Italy

**Keywords:** Guillain-Barré syndrome, COVID-19 vaccination, Bifacial weakness

## Abstract

Guillain-Barrè syndrome (GBS) is an acute immune-mediated neuropathy, possibly triggered by a recent infection or vaccination, and driven by an immune attack targeting the peripheral nervous system. GBS typically leads to ascending limb weakness, often with sensory and cranial nerve involvement 1–2 weeks after immune stimulation, but emergency and neurology physicians should be aware of its important clinical heterogeneity. In rare cases, bilateral facial nerve palsy can be the main clinical manifestation, as the case of the variant formerly known as bilateral facial weakness with paresthesias. An increasing number of case reports of GBS in patients receiving COVID-19 vaccination have been reported both during the pre-clinical phase and after large-scale authorities’ approval. We report two cases of bifacial palsy with paresthesias, a rare variant of GBS, both occurring after the first dose of COVID-19 vaccine Vaxzevria™ (formerly COVID-19 vaccine AstraZeneca), showing a favorable outcome after high-dose immunoglobulin therapy, and discuss the literature of GBS post-COVID-19 vaccination.

**Supplementary Information:**

The online version contains supplementary material available at 10.1007/s10072-022-05982-4.

## Introduction

Severe acute respiratory syndrome coronavirus 2 (SARS-CoV-2) infection has been associated with the development of autoimmune processes, such as anti-phospholipidic syndrome, immune thrombocytopenic purpura, and Guillain-Barrè syndrome (GBS) [1]. Molecular mimicry between the virus and human proteins has been suggested as a potential mechanism for these associations [2].

GBS can be triggered by respiratory or intestinal infections or by vaccination, being an episode of gastroenteritis caused by *Campylobacter jejuni*, influenza, and vaccination against influenza itself well-known causes of GBS. Clinical manifestations of the classic form of GBS include progressive, ascending, symmetrical flaccid limbs paralysis, along with areflexia or hyporeflexia with or without cranial nerve involvement, which can progress over days to weeks up to respiratory failure. Dysautonomic symptoms can be frequently reported [3]. Facial nerve palsy occurs in a significant number of cases of GBS, and can be observed in characteristic GBS variants, such as Miller-Fisher syndrome or pharyngeal-cervical-brachial weakness. In rare cases, bilateral facial nerve palsy can be the main clinical manifestation, as the case of the variant formerly known as bilateral facial weakness with paresthesias (BFP), whose incidence among all GBS manifestations is estimated to be about 1% [4].

A few small series or case reports of GBS occurring in patients with a recent history of COVID-19 have been published, but the link between COVID-19 and GBS remains to be investigated by case–control studies. As a general clinical aspect, GBS following COVID-19 is a sensori-motor variant whose manifestation can be independent from respiratory involvement. We can speculate that the molecular mimicry between SARS-CoV-2 spike protein and human proteins can trigger autoimmune diseases, including GBS [5–9].

The European Medicines Agency following the results of randomized, blinded, controlled trials approved four vaccines against COVID-19: two messenger RNA-based vaccines (Pfizer-BioNTech and Moderna); ChAdOx1 nCov-19 (AstraZeneca), a recombinant chimpanzee adenoviral vector encoding the spike glycoprotein of SARS-CoV-2; and Ad26.COV2.S (Johnson & Johnson/Janssen), a recombinant adenovirus type 26 vector encoding SARS-CoV-2 spike glycoprotein. DNA-based vaccines use adenoviruses whose replicative capacity has been eliminated and, additionally, have had the S protein’s DNA of the SARS-CoV-2 inserted.

An increasing number of case reports of GBS in patients receiving COVID-19 vaccination have been reported both during the pre-clinical phase and after large-scale authorities’ approval [9–12]. Here, we report two cases of GBS with facial bilateral palsy occurring after ChAdOx1-nCoV-19 vaccination and review the literature of GBS post-COVID-19 vaccination.

## Case report

### Patient 1

A caucasic 59-year-old woman, whose past medical history was remarkable only for acquired hypothyroidism due to Hashimoto thyroiditis, on Levothyroxine treatment, came to our observation because of acute onset of spontaneous burning pain of lower back, lower limb paresthesias, and bilateral facial weakness occurring 15 days after administration of the first dose of COVID-19 vaccine ChAdOx1 nCov-19. No fever, upper respiratory tract infection, or diarrhea was reported before or after vaccination.

Neurological examination demonstrated complete facial diplegia with Bell’s phenomenon and lagoftalm: she had bilateral loss of frontal forehead creases, could not raise her eyebrows, and could not whistle or smile. She had mild dysarthria due to facial diparesis with labial sounds. She had full strength in both upper and lower limbs; brisk upper limbs reflexes but lower limbs hyporeflexia. She had no objective sensory findings (VIDEO).

Computed tomography obtained when she was admitted to the emergency department was unremarkable. Cerebrospinal fluid examination (CSF) revealed clear fluid, normal opening pressure, glucose 59 mg/dl (normal range: 50–70 mg/dl), proteins 259 mg/dl (normal range: 15–45 mg/dl), and WBCs 1/mm^3, consistent with CSF albuminocytologic dissociation. The Meningitis/Encephalitis Panel (FILMARRAY™ multiplex PCR system bioMèrieux) on CSF was negative as well as the cerebrospinal fluid culture. RT-PCR testing for SARS-CoV-2 on CSF was negative. Diagnostic work-up for facial diplegia was completed by testing for antibodies against *Borrelia burgdoferI* on CSF and serum, paraneoplastic panel, Ab Anti-acethylcoline receptor, serological testing for autoimmune diseases, and serological tests for HIV, syphilis, cytomegalovirus, hepatitis B, hepatitis C, and herpes simplex virus. All these examinations did not report positive findings. Chest X-Ray was unremarkable.

Electrophysiological study revealed multifocal demyelinating sensorimotor (Table 1) polyradiculoneuropathy consistent with the diagnosis of GBS, AIDP variant, according to the electrodiagnostic criteria for classification of GBS [8]. Following Wakerley et al. [9] suggestions, the case was diagnosed as a variant of GBS with bifacial weakness with paresthesias (BFP), since the patient met all the proposed clinical diagnostic criteria for this variant. Intravenous immunoglobulins (IVIG) (0.4 g/kg per day) were administered along a 5-day period. Symptoms improved starting from the second day of IVIG treatment. The patient was discharged from hospital 2 days after the IVIG course was completed. She had slight movements of her facial muscles, and the distal paresthesias of his lower extremities were reduced.

**Table 1:** Electrophysiological study in patients 1 and 2

Nerve	Latency (ms)	Amplitude (mV)	MCV(m/s)	SCV (m/s)
Right facial	Patient 1. Unexcitable Patient 2. Unexcitable	Patient 1. Unexcitable Patient 2. Unexcitable	Patient 1. Unexcitable Patient 2. Unexcitable	
Left facial	Patient 1. Unexcitable Patient 2. Unexcitable	Patient 1. Unexcitable Patient 2. Unexcitable	Patient 1. Unexcitable Patient 2. Unexcitable	Patient 1. Unexcitable Patient 2. Unexcitable
Right median	Patient 1. 7.2 (F wave: 48.8) Patient 2. Missing data	Patient 1. 8.1 Patient 2. Missing data	Patient 1. 30.1 Patient 2. Missing data	Patient 1. 29.2 Patient 2. Missing data
Right ulnar	Patient 1. 4.14 (F wave: 46.4) Patient 2. 4.72 (F wave: 44.8)	Patient 1. 6.2 Patient 2. 11.5	Patient 1. 31 Patient 2. 42.8	Patient 1. 27.2 Patient 2. 34.5
Left median	Patient 1. 6.96 (F wave: 41.8) Patient 2. 7.44 (F wave: 53.9)	Patient 1. 6.6 Patient 2. 10.5	Patient 1. 29.9 Patient 2. 35.6	Patient 1. 29.2 Patient 2. 49.3
Left ulnar	Patient 1. 4.31 Patient 2. 4.78	Patient 1. 6.4 Patient 2. 12.1	Patient 1. 31.3 Patient 2. 37.4	Patient 1. 31.8 Patient 2. 36.7
Right deep peroneal	Patient 1. 8.86 (F wave: absent) Patient 2. 8.80 (F wave: 76.5)	Patient 1. 2.9 Patient 2. 5.5	Patient 1. 34.4 Patient 2. 39.5	
Left deep peroneal	Patient 1. 8.26 Patient 2. 7.58	Patient 1. 3.6 Patient 2. 6.2	Patient 1. 25.5 Patient 2. 36.0	Patient 1. Missing Data Patient 2. 53.0
Right tibial	Patient 1. 11 (F wave: 75.5) Patient 2. 7.34 (F wave: 78.5)	Patient 1. 4.9 Patient 2. 8.5	Patient 1. 31.3 Patient 2. 37.4	
Left tibial	Patient 1. 9.79 (F wave: 76.7) Patient 2. 7.35 (F wave: 74.2)	Patient 1. 5.5 Patient 2. 9.1	Patient 1. 32.2 Patient 2. 40.4	

*MCV*, motor conduction velocity; *SCV*, sensitive conduction velocity.

### Patient 2

A Caucasian 43‐year‐old male previously healthy patient with an uneventful medical history was admitted to our department with a subacute onset of facial pain and numbness with weakness of eye closure, observed 7 days after administration of COVID-19 vaccine first dose ChAdOx1. During the subsequent 7 days, he started suffering from lower limbs and hands paresthesias. Neurologic examination at the time of our observation revealed facial diplegia with left-side prevalence; symmetrical weakness with Medical Research Council (MRC) grade 4 + out of 5 in distal muscle groups of both upper and lower limbs; normal upper limbs tendon reflexes but lower limbs hyporeflexia; no sensory deficit was observed.

Cerebrospinal fluid examination (CSF) revealed clear fluid, normal opening pressure, glucose 71 mg/dl (normal range: 50–70 mg/dl), proteins 200 mg/dl (normal range: 15–45 gm/dl), and WBCs 6/mm^3, consistent with CSF albuminocytologic dissociation examination. Screening for other infectious, autoimmune, metabolic, or systemic diseases, as performed for patient N. 1, was unremarkable. Unfortunately RT-PCR for SARS-CoV-2 virus on CSF was not performed in this case. Diagnostic criteria for bifacial weakness with paresthesias variant of GBS were fulfilled and the patient started on the second day from admission intravenous immunoglobulin at the dosage of 0.4 g/kg per day for 5 days. After 8 days, he showed significant improvement of facial strength and reduction of paresthesias. During hospitalization, he had a single episode of atrial fibrillation, solved with pharmacological cardioversion with flecainide, interpreted as a disautonomyc manifestation of GBS.

Electrophysiological study revealed multifocal demyelinating sensorimotor polyradiculoneuropathy (Table 1), with prevalent involvement of lower limbs compatible with the diagnosis of Guillain-Barré syndrome (GBS), AIDP variant, according to electrodiagnostic criteria for classification of GBS (8), with a mid CMAP reduction in lower limbs.

## Discussion

Our study reports 2 cases of BFP occurring within 2 weeks after the first dose of ChAdOx1 nCov-19 vaccine was administered. The link between such COVID-19 vaccine and BFP is suggested by the temporal association between vaccine administration and clinical manifestations of BFP and by the lack of other known factors able to trigger GBS.

About two-third of patients affected by GBS report symptoms suggesting a recent or ongoing infection, such as fever or gastrointestinal and upper respiratory tract manifestations. A definitive infection can be identified in half of the cases suffering GBS and COVID-19 itself has been shown to be able to trigger GBS based on epidemiological investigations [13]. Filosto et al. [12] reported an increase in GBS incidence during the COVID-19 outbreak in Northern Italy, supporting a pathogenic link (rate of 47.9 cases of GBS per 100 000 COVID-19 infections). Such link between SARS-CoV-2 infection and GBS was confirmed by a systematic review and meta-analysis of observational cohorts and case series, which highlighted the association between COVID-19 and GBS, reporting a high percentage of demyelinating GBS variant and estimating GBS prevalence as 15 cases per 100,000 SARS-CoV-2 infections [14, 15]. We also found 5 case reports in literature of facial diplegia in COVID-19 infection context [16–19].

Based on biological plausibility and temporal association, vaccines have been suggested to increase GBS risk. Vaccine-associated GBS is defined by a characteristic clinical syndrome suggesting an acute immunomediated polyneuropathy within the 6-week period after vaccine administration [20]. Most of the data about the link between vaccination and GBS regards patients receiving influenza vaccine. In 1976, a sevenfold increase of GBS cases was noticed in the USA during the national H1N1 swine flu vaccination program. Moreover, a study investigating GBS incidence during the 1992–1993 and 1993–1994 vaccination programs reported that influenza vaccine was associated with a 1.7-fold increase of GBS risk, as compared to unvaccinated population [21, 22]. In any case, an extensive review of the literature found a post-influenza vaccination risk expressed as a hazard ratio (HR) of 1.11 (95% CI: 0.51–2.43), which was lower than observed after influenza infection (HR = 4.89, 95% CI: 1.17–20.36). Evidence regarding other vaccines is extremely limited although a number of case report highlights a strict time relationship between vaccination and GBS [20, 23, 24].

Looking at COVID-19 vaccination campaign, the Food and Drug Administration (FDA) has announced that 100 cases of GBS have been reported after the administration of approximately 13 million doses of Johnson & Johnson’s COVID vaccine, deciding to include GBS among the potential side effects (https://www.fda.gov/media/150723/downlad). The European medicine agency (EMA) did the same for the AstraZeneca vaccine (https://www.ema.europa.eu/en/documents/covid-19-vaccine-safety-​update/covid-19-vaccine-safety-update-vaxzevria-previously-covid-19-vaccine-astrazeneca-8-september-2021_en.pdf).

A systematic literature review performed by searching the Pubmed and Scopus databases with the search string “Guillain-Barrè Syndrome and COVID vaccination” retrieved 78 articles: 67 did not report data on patients with GBS following COVID-19 vaccination and 11 were found to report data on GBS cases following COVID-19 vaccination. Table 2 reports the main clinical characteristics of the 2 patients observed and of the 19 patients retrieved by the literature search. Bilateral facial palsy was reported in 15 patients, and all patients reported albuminocytological dissociation. Two studies reported small clusters “of an unusual variant of Guillain–Barre syndrome” following the vaccination. First study reported 4 cases of BFP GBS variant occurring 11 to 22 days after administration of the first dose of AZV were reported [25]; the second report described seven cases of BFP GBS variant syndrome occurring within 2 weeks of the first COVID-19 vaccine [26].

**Table 2:** Features of GBS cases post-vaccination

Demographics	Co-morbidities	Vaccine	Onset of symptoms in relation to vaccination	Clinical presentation	CSF	NCS	Therapy
Age: 65 Sex: Female Ethnicity: Caucasian **(Patient 1)**	Hypothyroidism due to Hashimoto thyroiditis	AZ	2 weeks post-1st dose	- Burning pain of lower back—Lower limb paresthesia - Bilateral facial weakness	- Proteins: 259 mg/dl - WBCs: 1/mm^3	Multifocal demyelinating sensorimotor polyradiculoneuropathy	Intravenous immunoglobulin at 0.4 g/kg per day, for 5 days
Age: 43 Sex: Male Ethnicity: Caucasian **(Patient 2)**	None	AZ	1 week post-1st dose	- Onset of bifacial pain and numbness with weakness of eye closure - Lower limbs and hands paresthesias	- Proteins: 200 mg/dl - WBCs: 6/mm^3	Multifocal demyelinating sensorimotor Polyradiculoneuropathy with prevalent involvement of lower limbs	Intravenous immunoglobulin at 0.4 g/kg per day for 5 days
Age: 51 Sex: Male Ethnicity: Caucasian [28]	Non-ST segment elevation myocardial infarction	AZ	2 weeks post-1st dose	Nadir: at 2 weeks - Lower back pain - Bifacial weakness - Dysphagia - Diplopia - Respiratory failure - Upper limb motor deficit - Progressive ascending lower limb sensorimotor deficit - Areflexia	- Protein: 70 mg/dl - WBCs: 0/mm^3	Absent ulnar and sural sensory responses with slowed median sensory and motor NCV, prolonged distal motor latencies, absent F waves	Intravenous immunoglobulin
Age: 65 Sex: Female Ethnicity: Caucasian [28]	None	AZ	Onset: 1 week-post 1st dose	Nadir: at 2 weeks - Lower back pain - Dysphagia - Diplopia - Respiratory failure - Progressive ascending upper limb and lower limb sensorimotor deficit - Areflexia	- Protein: 251 mg/dl - WCC: 5 mm^3	Absent upper limb sensory responses with sural sparing, prolonged distal latencies, reduced amplitudes with temporal dispersion, slowed motor NCV, absent F waves	Intravenous immunoglobulin
Age: 66 Sex: Male Ethnicity: Caucasian [28]	Renal cell carcinoma Atrial fibrillation Hypercholesterolemia	AZ	3 weeks post-1st dose	Nadir at 4 weeks: - Lower back pain - Progressive ascending sensorimotor involvement - Proximal lower limb weakness - Areflexia	- Protein: 150 mg/dl - WCC: 0	Absent median sensory responses with sural sparing, absent peroneal and tibial motor responses, prolonged median distal motor, and F wave minimum latencies with slowed motor NCV	Intravenous immunoglobulin
Age: 64 Sex: Female Ethnicity: Caucasian [29]	Hypertension Diabetes mellitus type 2 Hyperlipidemia	Ad26.COV2.S	2 weeks post-vaccination	- Headache in the frontal and periorbital regions - Ageusia and hyposalivation - Dysarthria, dysphagia, dysphasia - Bilateral facial weakness	- Protein: 302 mg/dl - WCC: 0	Missing data	IVIG and plasmapheresis
Age: 38 Sex: Male Ethnicity: Caucasian [30]	Anxiety and depression	Ad26.COV2.S	2 weeks post-vaccination	- Onset with numbness and tingling in his tongue, lips, and bilateral hands and feet - Facial weakness, slurred speech - Bilateral hand and foot paresthesias - 30 days after vaccination: difficulty moving his mouth and forming words, as well as difficulty drinking from a straw and controlling his lips, cheeks, and tongue while eating	- Protein: 181 mg/dl - WCC: 7 mm^3	Missing data	Intravenous immunoglobulin (IVIG) over 2 days to a total dose of 2 g/kg
Age: 37 Sex: Male Ethnicity: Caucasian [31]	None	AZ	14 days after 1st dose	- Lower back pain - Symmetrical progressive ascending upper limb and lower limb sensorimotor deficit - Areflexia	- CSF protein: 177 mg/dl - WCC: 0	EMG: patchy attenuation of the upper limb motor responses NCS: demyelinating neuropathy	Intravenous immunoglobulin (IVIg) with a dose of 2 g/kg daily for a total duration of 5 days
Age: 67 Sex: Male Ethnicity: Caucasian [32]	None	AZ	15 days after 1st dose	- Bifacial weakness - Progressive bilateral lower limb motor deficit - Areflexia	- Protein: 390 mg/dl - WCC: 0	Demyelinating neuropathy	Missing data
Age: 62 Sex: Female Ethnicity: Caucasian [33]	Bronchiectasis, asthma, osteoporosis, and migraine	AZ	11 days after 1st dose	Ascending bilateral lower limb weakness preceded by paraesthesia and numbness	- Protein: 90 mg/dl - WCC: 1	EMG: demyelinating, sensorimotor polyneuropathy	Intravenous immunoglobulin at a dose of 2 g/kg body weight divided over 5 consecutive days
Age: 54 Sex: Male Ethnicity: Caucasian [25]	None	AZ	16 days post-first dose	- Distal dysesthesia in his feet and hands, which ascended over 2 days, but had begun to recede as facial weakness developed	- Protein: 160 mg/dl - WCC: 17 mm^3	Demyelinating neuropathy	Oral prednisolone 60 mg for 5 days
Age: 20 Sex: Male Ethnicity: Iranian [25]	Ulcerative colitis	AZ	26 days post-first dose	- Occipital headache, dysesthesia in his distal lower limbs and facial diplegia	- Protein: 123 mg/dl - WCC: 14 mm^3	Demyelinating neuropathy	Oral prednisolone 60 mg for 5 days
Age: 57 Sex: Male Ethnicity: Caucasian [25]	Asthma and osteoarthritis requiring bilateral knee replacements	AZ	21 days post-first dose	- Lumbar back pain that radiated into his flanks - After 4 days dysarthria and facial weakness - Distal dysesthesia in his feet and proximal leg weakness that continued to progress until admission	- Protein: 247 mg/dl - WCC: 8 mm^3	Facial NCS and electromyography were not performed. Sensory and motor NCS were normal in the upper and lower limbs. Minimum F wave latencies were 26 to 33 ms in the median nerves	Missing data
Age: 43 Sex: Female Ethnicity: Caucasian [26]		AZ	10 days post-first dose	- Facial diplegia - Motor strength - MRC grade 1/5 in UL and LL - Areflexia - Respiratory failure	- Cells: 2/mm^3^ (normal < 5 cells/mm^3^) - Protein: 85 mg/dl (normal 15–45 mg/dl)	NCS: Demyelinating neuropathy (delayed distal motor latencies, slowing of conduction velocity) prolonged F waves, absent sensory nerve action potentials)	IVIg
Age: 67 Sex: Female Ethnicity: Caucasian [26]		AZ	14 days post-first dose	- Right abducens palsy, facial diplegia, and bulbar palsy—Distal sensory impairment in the legs (pinprick) - MRC grade 1/5 power in all limbs - Areflexia - Respiratory failure	- Cells: 3/mm^3^ - Protein: 345 mg/dl)	NCS: axonal motor — sensory neuropathy (reduced compound motor action potentials, absent F waves, absent sensory nerve action potentials)	IVIg
Age: 63 Sex: male Ethnicity: missing data [37]	None	AZ	9 days following his first dose	- Onset with lower back pain - 5 days later: severe bilateral facial weakness, unsteadiness, lower limb weakness, and paraesthesia over a 48-h period - On his second day of admission, diplopia on lateral gaze bilaterally, consistent with partial cranial nerve III palsies	- Cells: 5/mm^3^ - Protein: 299 mg/dl	- Long-standing axonal neuropathy with reduced motor and sensory amplitudes - Length-dependent chronic neurogenic changes on EMG, but no acute abnormalities	IVIg
Age: 61 Sex: Female Ethnicity: missing data [36]	Multiple sclerosis	AZ	10 days after first dose	- Bifacial, left > right, weakness with prominent lower facial involvement - Asymmetrical, left > right, lower limb weakness (MRC 3/5 proximally and 4/5 distally) - Tingling in her feet and hands	- Cells: acellular - Protein: 164 mg/dl	- Motor NCS fulfilled the criteria for demyelinating polyneuropathy - Sensory NCS were within normal limits - No acute denervation potentials on needle electromyogram (EMG) studies	IVIg
Age: 56 Sex: male Ethnicity: missing data [36]	None	AZ	A week later first dose	- Sudden‐onset severe back and lower limb radicular pain—Waist down numbness and a sensation of heaviness in his legs - Tingling and numbness in his fingertips—Bilateral lower motor neuron facial weakness - Decreased vibration sensation at the ankles - Areflexia	- Cells: two lymphocytes only - Protein: 160 mg/dl	- Motor NCS fulfilled the criteria for demyelinating polyneuropathy - Sensory NCS were within normal limits - No acute denervation potentials on needle electromyogram (EMG) studies	IVIg
Age: 41 Sex: male Ethnicity: missing data [34]	Obesity	Ad26.COV2.S	15 days after vaccination	- Onset with left-sided facial droop - After 7 days: bilateral facial palsy, subjective weakness, and paresthesias in all extremities (motor strength was 4 + /5 (Medical Research Council grade) in all four extremities - Deep tendon reflexes were absent bilaterally at the patella and the Achilles with mute plantar responses	- Cells: 50 mm^3^ - Protein: 562 mg/dl)	Prolonged distal latency with conduction block and slow conduction velocity in bilateral tibial, peroneal nerve, and absent F waves were supportive for demyelinating GBS	IVIg
Age: 59 Sex: male Ethnicity: Caucasian [35]	Hypertension and hyperuricemia	AZ	10 days after vaccination	- Gait ataxia - Global areflexia - Distal paraesthesia both at the lower and upper limbs - Segmental strength was diffusely preserved (MRC: 5/5) - bilateral facial palsy	- Cells: reported as normal - Protein: 140 mg/dl)	Diffuse and evident signs of motor nerve demyelination (upper and lower limbs, and cranial district)	IVIg
Age: 53 Sex: Female Ethnicity: missing data [38]	No relevant medical history	AZ	13 days after first dose	Bilateral facial palsy Tetramelic distal paresthesias Progressive minimal limbs weakness	Cells: 4 leukocytes/mm^3^ Protein: 96 mg/dl	EMG: a microvoltage pattern with reinnervation of orbicularis oris	Five double filtration plasmapheresis session within 10 days

*CSF*, cerebrospinal fluid; *NCS*, nerve conduction study; *AZ*, AstraZeneca; *Ad26.COV2.S*, Johnson & Johnson

We also searched Pubmed and Scopus databases with the search string “facial diplegia covid vaccination,” founding other two case reports in which clinical, electrophysiological, and laboratoristic data were compatible with Guillain-Barrè syndrome diagnosis [34, 35].

Facial diplegia or bilateral facial nerve palsy (B-FNP) is rare with an incidence of just 1 per 500,000 population and only 20% cases are idiopathic [27].

Interestingly a viral vector vaccine (both Johnson & Johnson and Astrazeneca) was administered to 14/15 patients observed, including both our cases and those reported by literature. Notably, as reported worldwide, the highest risk of GBS after vaccination is attributed to flu virus-based vaccination, either a live attenuated or inactivated influenza vaccine. There is evidence that adenovirus-vector might induce higher levels of specific T cells, whereas mRNA vaccines might induce higher antibody titers [39]. We also know that cell-mediated autoimmune mechanisms may be relevant in the pathogenesis of Bell’s palsy, as demonstrated by the elevated concentrations of the cytokines interleukin-1 (IL-1), IL-6, and tumor necrosis factor-alpha (TNF-alpha) in patients with Bell’s palsy, compared with control populations, suggesting an activation of cell-mediated effectors [40]. Anyway, the pathophysiology of the BFP and the link between this clinical and vaccination against COVID-19 still remain unclear and require further research.

## Conclusion

This study warrants early recognition and treatment through active surveillance for GBS after COVID-19 vaccination, with specific focus on the rare bilateral facial palsy as a main symptom of GBS. Future research should aim to determine the predisposing host factors and biological mechanisms underlying this association and the high frequency of facial nerve involvement. Perhaps the SARS-CoV-2 antigen or chimpanzee adenovirus adjuvant contained in the vaccination may induce immune mechanisms leading to neuropathy for cross-reaction between antibodies against the spike protein and peripheral nerve constituents.

## Supplementary Information

Below is the link to the electronic supplementary material.

Supplementary file1 (MP4 50373 KB)
